# Orthopaedic research in low-income countries: A bibliometric analysis of the current literature

**DOI:** 10.1051/sicotj/2019038

**Published:** 2019-11-26

**Authors:** Simon Matthew Graham, Ciaran Brennan, Maritz Laubscher, Sithombo Maqungo, David G. Lalloo, Daniel C. Perry, Nyengo Mkandawire, William J. Harrison

**Affiliations:** 1 Liverpool School of Tropical Medicine Liverpool L3 5QA UK; 2 Orthopaedic Research Unit, Department of Orthopaedic Surgery, Groote Schuur Hospital, University of Cape Town Cape Town 7700 South Africa; 3 Orthopaedic Trauma Service, Groote Schuur Hospital, University of Cape Town Cape Town 7700 South Africa; 4 Director, Liverpool School of Tropical Medicine Liverpool L3 5QA UK; 5 Alder Hey Hospital Liverpool L12 2AP UK; 6 Oxford Trauma, NDORMS, University of Oxford OX3 9DU UK; 7 University of Malawi College of Medicine Private Bag 360 Chichiri, Blantyre 3 Malawi; 8 Countess of Chester Hospital Chester CH2 1UL UK; 9 AO Alliance Foundation, Africa Regional Director Davos Switzerland

**Keywords:** Bibliometric analysis, Low-income countries, Orthopaedic, Research

## Abstract

*Background*: To perform a bibliometric analysis and quantify the amount of orthopaedic and trauma literature published from low-income countries (LICs).

*Methods and methods*: The Web of Science database was utilised to identify all indexed orthopaedic journals. All articles published in the 76 orthopaedics journals over the last 10 years were reviewed, to determine their geographic origin.

*Results*: A total of 131 454 articles were published across 76 orthopaedic journals over the last 10 years. Of these, 132 (0.1%) were published from LICs and 3515 (2.7%) were published from lower middle-income countries (LMICs); 85.7% (*n* = 112 716) of published orthopaedic research was undertaken in a high-income setting. The majority of the studies (*n* = 90, 74.4%) presented level IV evidence. Only 7.4% (*n* = 9) were high-quality evidence (level I or II). Additionally, the majority of research (74 articles, 56%) was published in partnership with high-income countries (HICs).

*Conclusions*: There is a stark mismatch between the publication of scientific reports on orthopaedic research and the geographical areas of greatest clinical need. We believe there is an urgent need for orthopaedic research to be carried out in low-income settings to guide treatment and improve outcomes, rather than assuming that evidence from high-income settings will translate into this environment.

*Level of evidence*: IV

## Introduction

Musculoskeletal disease represents a large proportion of the burden of disease in low- and middle-income countries (LMICs) but is often a neglected issue that goes untreated [1]. It is estimated that more than 90% of injury-related deaths worldwide occur in these countries [2], accounting for approximately the same number of deaths as malaria, tuberculosis and human immunodeficiency virus/acquired immunodeficiency syndrome (HIV/AIDS) combined [3]. By 2020, it is expected that 7 out of 10 deaths in LMICs will be as a result of non-communicable disease, with road traffic accidents rising to the third leading cause of death [4].

Considering that the burden of musculoskeletal disease, which commonly goes untreated [5] in low-income countries (LICs), there is an apparent lack of orthopaedic research originating from these settings and the contribution of LICs to peer-reviewed literature on the topic appears to be negligible [6].

The rates of pre-hospital death due to trauma are also highest in countries with the fewest resources [7]. There is an urgent need for trauma research in LMICs, in order to understand the specific issues in these settings and improve outcomes. Additionally, little is known about the outcomes of non-trauma-related orthopaedic surgery and pathology, such as hip or knee replacements, in these countries.

A recent study [8], looking at worldwide publications in fracture surgery, found that only 0.1% of papers were published from LICs over a 10-year period, with an overwhelming majority (86.6%) from high-income countries (HICs).

Management of musculoskeletal disease in HICs is guided by evidence-based practice and there is a growing body of research to support this [9]. However, there is a disparity between LICs and HICs when it comes to resources and training [7, 9]. This often means LICs rely upon evidence from research in HICs, which have a very different patient demographic, available resources and disease burden that are commonly not translatable to a low-income setting. Furthermore, clinical conditions with management guidelines based on the high-income context present a challenge to clinicians in low-resource settings, who are likely to feel vulnerable when resource constraints force clinical decisions that are different from published protocols and treatment strategies that are likely to be contextually irrelevant [10].

The aim of this paper was to ascertain the number and proportions of peer-reviewed articles being published in orthopaedic journals across all sub-specialities within orthopaedic surgery that originate from LICs.

## Materials and methods

The “Clarivariate Analytics” Web of Science database was searched to identify all indexed articles from LICs published in orthopaedic journals over the last 10 years (January 2007 to September 2017). Included articles were limited to English language publications. Web of Science was used, as it has a facility to filter journal titles to include only English language orthopaedic journals.

Articles were included if one or more authors listed on the publication was from a LIC. The research material origin was not part of the inclusion criteria. This was defined according to a country’s status as appearing on the World Bank Classification for the current fiscal year (2018) [11]. As of 1 July 2018, low-income economies are defined as those with a Gross National Income (GNI) per capita of $995 or less in 2017; lower middle-income economies are those with a GNI per capita between $996 and $3895; upper middle-income economies are those between $3896 and $12 055; high-income economies are those with a GNI per capita of $12 055 or more. Articles that were letters or correspondence were excluded.

For the purposes of this article, countries appearing on the database under their former names were included under their current name; for example, one journal listed “Zaïre”, which was included as per the World Bank Classification as “Democratic Republic of the Congo”.

For every article included, the following information was extracted: journal title, geographic location and country, year of publication, research subject, number of citations and level of evidence. Using this information, the number of articles from LICs, LMICs, upper middle-income countries (UMICs) and HICs was determined. The level of evidence was established by reviewing the abstracts and or full texts of articles and then categorised according to the criteria established in *Journal of Bone and Joint Surgery American Volume* [12].

The nature of the research was categorised by two orthopaedic surgeons (first and second authors), according to the main focus of the article, which was determined following review of the full texts. If an article had more than one focus, it was listed under the category “Multiple focus”. Articles relating to correction of deformity were included under the heading “Limb reconstruction”, unless their predominant focus was paediatric orthopaedics, such as correction of club foot, in which case they were included under the heading “Paediatric”. The same orthopaedic surgeons jointly decided on the level of evidence of the studies included.

Articles were categorised under the heading “Other” if they were economic analyses or commentary articles about services or training.

## Results

There are 76 orthopaedic journals listed on the Web of Science database. Across these journals, a total of 131 454 articles published since 2007 were identified that met the inclusion criteria.

A total of 132 articles from LICs were identified. Of these, three were subsequently excluded (2 letters and 1 article that was subsequently retracted). On reading the full articles, one further study was excluded as it was only available in German.

The search strategy used meant that the results generated were based upon the country of origin for an article rather than the article itself; therefore seven further results were excluded due to the fact that they were duplicate articles carried out in more than one LIC.

Two articles in *International Orthopaedics* were research collaborations with data from Haiti, Afghanistan and Zaïre. They therefore appear as 2 articles out of the 121 final articles included in the study, but count for three countries in the list of the LICs producing orthopaedic research.

This was also the case for an article in *Archives of Osteoporosis,* which was a research collaboration between Uganda and Zimbabwe counting for 1 article out of the 121 total but two LICs, along with two further articles in *Orthopaedics & Traumatology: Surgery and Research,* that were found to have also been conducted across two different LICs.

**Figure 1:**
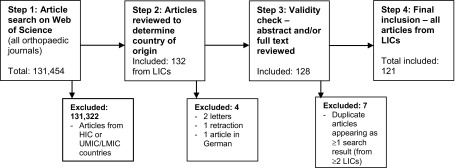
Flow diagram of article-selection process.

For all of these articles from more than one LIC, all relevant countries were included in the overall results, but the paper was recorded as only one article. This resulted in a higher number of total countries (*n* = 128) compared to the overall number of articles (*n* = 121) (refer to Figure 1 for a flow diagram of the article-selection process).

**Figure 2:**
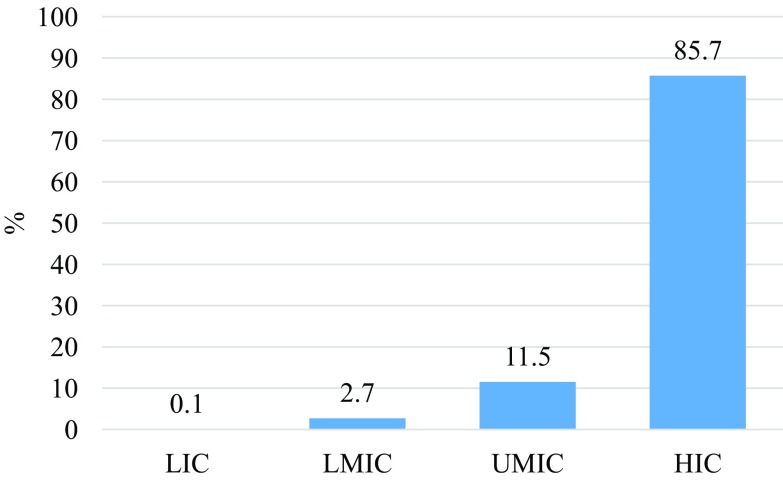
Percentage of orthopaedic publications according to country income level.

This gave a total of 121 (0.1%) articles originating from 20 different LICs; 3515 (2.7%) were published from LMICs and the majority of articles, 112 716 (85.7%) were published from HICs (Figure 2).

Forty-one orthopaedic journals had no published articles from LICs. The journal with the highest number of publications from LICs was *Injury*, which had published 20 articles from this economic setting over the last 10 years. After *Injury,* the orthopaedic journals with the most publications from LICs in the last 10 years were *Chirurgie de la Main* (*n* = 16)*, International Orthopaedics* (*n* = 11), *Clinical Orthopaedics and Related Research* (*n* = 8) and *Orthopaedics & Traumatology – Surgery & Research* (*n* = 8) respectively (see Table 1).

**Table 1:** Top five orthopaedic journals with most publications coming from LICs, 2007–2017.

Journal	Number of articles
*Injury*	20
*Chirurgie de la Main*	16
*International Orthopaedics*	11
*Clinical Orthopaedics and Related Research*	8
*Orthopaedics & Traumatology – Surgery & Research*	8

The most common geographic origin of the studies was sub-Saharan Africa (*n* = 91/128, 71.1%); however, the LIC with the most publications was Nepal with 25 articles (19.5%). Details of the geographic origins of the articles and the LICs from which they were published can be found in Tables 2 and 3, respectively.

**Table 2:** Geographic origin of articles from LIC by region, 2007–2017. The total number of countries is 128 because 5 of 121 articles were studies conducted across more than one LIC.

Geographic area	Number of articles
Sub-Saharan Africa	91
Asia	33
Caribbean	4

**Table 3:** Geographic origin of articles from low-income countries by country, 2007–2017.

Country	Number of articles
Nepal	25
Malawi	17
Senegal	14
Uganda	13
Afghanistan	8
Ethiopia	8
United Republic of Tanzania	7
Benin	5
Burkina Faso	5
Togo	5
Haiti	4
Zimbabwe	4
Madagascar	3
Sierra Leone	3
Democratic Republic of the Congo	2
Gambia	1
Liberia	1
Mali	1
Niger	1
Rwanda	1

**Figure 3:**
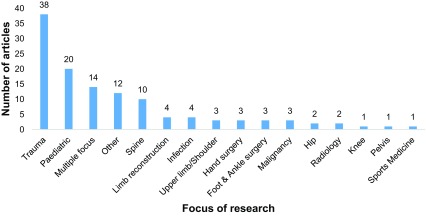
Number of articles (*n* = 121) for each area of research, 2007–2017.

The most common focus of research was trauma (*n* = 38, 31.4%) followed by paediatric orthopaedic research articles (*n* = 20, 16.5%). A breakdown of the focus of research for all articles is detailed in Figure 3. Nearly all of the studies (*n* = 91, 75.2%) presented level IV evidence. Only 7.4% (*n* = 9) were high-quality evidence (level I or II).

After reviewing the full texts and the authors’ affiliations for each article, it was found that only 41 of the 121 articles (33.9%) were written solely by authors from LICs. The remaining 80 articles had authors from both LICs and HICs or MICs. Of those 80, 92.5% (*n* = 74) were authors from HICs. The HICs most frequently collaborating with research from LICs are detailed in Table 4.

**Table 4:** Top high-income countries collaborating with research in low-income countries, 2007–2017.

High-income country	Number of articles
USA	16
UK	6
Norway	5
Japan	4
Canada	3
Belgium	3

## Discussion

The Global Surgery 2030 report by the Lancet Global Commission [10] states that approximately 30% of the global burden of disease is attributable to surgically treatable conditions and that 5 billion people are unable to access surgical services worldwide – predominantly people in LMICs. Trauma is a major cause of death in these settings [7] and although orthopaedic surgeons in LICs are facing an increasing trauma pandemic [13], the research presented in this paper demonstrates that only 0.1% of all publications in orthopaedic journals over the last 10 years originate from LICs. This confirms that there is little research to guide management and develop clinically and economically appropriate treatment methods with the aim of improving outcomes in LICs.

More than half of all the orthopaedic journals on the Web of Science database had not published any articles from LICs and more than half of the articles originating from LICs were published in just five of the orthopaedic journals. Of the 35 orthopaedic journals listed on Web of Science that had published articles from LICs, 21 (60%) had published only one or two articles over the last 10 years from a country in this setting. Thus, the majority of orthopaedic journals are not consistently publishing research carried out in LICs. One of the main reasons for this may be that research originating from LICs is of low quality – the majority of the papers published from LICs were level IV evidence (*n* = 90, 74.4%) and only 7.4% (*n* = 9) were level I or II. Furthermore, it may be a sign that there is a simple lack of orthopaedic research being undertaken in LICs.

According to latest World Bank data [14], the total number of people living in LICs is estimated at 659 272 676, equating to 8.86% of the estimated total world population. The World Bank data also estimate the number of people living in HICs to be 1.19 billion (15.99%). Therefore, despite the fact that almost 84% of the total world population lives in LMICs, our research shows that 85.7% of published orthopaedic research is being carried out in a HIC setting. Less than 0.1% of research is taking place in a LIC, despite 8.86% of the population living in these countries. This shows that current research is heavily biased towards a HIC demographic and is not truly reflective of the needs of the whole world population.

Interestingly, the present study found that approximately one third of articles published from LICs across all orthopaedic journals in the last 10 years were written by authors solely from LICs and that the remainder were co-authored by researchers with affiliations in a HIC or MIC. This reflects what appears to be a common trend of researchers in HIC countries collaborating with their counter parts in a LICs setting. There are many positives related to country collaborations, including the opportunity for LIC to have access to research training and experience that they would not otherwise have access too. However, the lack of independent articles published solely by authors from LICs is very apparent.

Nearly one fifth (19.5%, *n* = 25) of the articles published from LICs originated from Nepal. Of note, more than half (56%, *n* = 14) of these articles originating from Nepal involved collaboration with a HIC/MIC and nine articles were co-authored with researchers from the USA. Academic collaboration between HICs and LICs, such as the model in Nepal, is a potential approach in assisting LICs to consolidate and augment their research strategy, approach and output.

We have also had success using this approach in LIC. An example of this is the Malawi National Joint Registry, funded by United Kingdom charity donors. This collaboration is addressing important clinical problems in Malawi using local resources, researchers and institutes, in collaboration with funding and research expertise from the United Kingdom. This approach has resulted in numerous publications in international journals and recognition in The Global Surgery 2030 report by the Lancet Global Commission [10] as a model of future health care in a LIC [15–17].

Only 10% of the 132 publications from LICs were classified as level I or II evidence, with the majority of articles being case series or level IV evidence. Our findings mirror that of a recent study reported by Wu et al. [18], who found that only 10% of the studies from LMICs evaluated reported level I or II evidence but that when investigators in LMICs and from HICs collaborated, research was of a higher evidence quality [18]. The present study similarly found that of the nine articles that were classed as high-level evidence, 78% (*n* = 7) were collaborative articles. Six articles were collaborations between LICs and HICs and the remaining article was a LIC/MIC collaboration between Ethiopia and South Africa, highlighting the positive benefits of collaboration between two different settings.

Reasons for this apparent lack of research are multi-factorial and largely attributable to the shortage of human and financial resources for orthopaedic research and the lack of skills and incentives to conduct it. Additionally, there are also a number of logistical issues. For example, patients commonly do not have the financial means to attend follow-up or simply live too far from their primary hospital to attend any follow-up appointments. This results in high lost-to-follow-up rates and small overall outcome sample sizes – a problem that few, if any, journals have sympathy for when it comes to the publication of research. The UN Educational, Scientific, and Cultural Organization (UNESCO) estimates that only 13% of the world’s scientists are located in Africa, Latin America and the Middle East, reflecting the global issue of lack of research and scientific expertise in LICs [19].

The Lancet Commission of Global Surgery 2030, reported similar findings to the present study in their bibliometric analysis focusing on the relative volume of surgical research output between 2009 and 2013 from authors of four country groups: high-income, upper middle-income, lower middle-income, and LICs. Of the 35 countries with the highest volumes, HICs had the greatest presence with 264 458 (85%) reports, followed by UMICs with 37 838 (12%) reports and LMICs with 8371 (3%) reports. Multi-country collaboration on surgical research within income groups was low, leading to the conclusion that the highest volume of surgical research is not done in, or by, the countries with greatest clinical need. Rather, the volume of surgical research output correlates with total GDP [10].

Historically, global health research efforts have not focused on diseases with the highest burden or on regions with the greatest clinical need [20, 21, 22]. With approximately 36.9 million people living with HIV worldwide, 25 million of whom are in areas in sub-Saharan Africa, the majority of research and research funding in HIV is based in LICs [10, 23]. It is estimated that more than 90% of injury-related deaths worldwide occur in low and LMICs [2], accounting for approximately the same number of deaths as malaria, tuberculosis and HIV/AIDS combined [3]. By 2020 it is expected that 7 out of 10 deaths in LMICs will be as a result of non-communicable disease, with road traffic accidents rising to the third leading cause of death [4]. The research we have presented in this paper shows that this is not reflected in a higher proportion of research on injury-related deaths emanating from LMICs.

## Limitations

A limitation of the study is the fact that the methodology for searching the Web of Science database selected solely orthopaedic journals and therefore any articles in journals not listed on their database as an orthopaedic journal would not have been included in the search results. This includes any orthopaedic publication in non-orthopaedic journals, such as the *Lancet*. Furthermore, in clinical practice and in literature from LICs, trauma and orthopaedics are often grouped with other surgical specialities, and publications, such as the *Tropical Doctor* and *East and Central African Journal of Surgery*, are not included on the Web of Science database. This may have led to an underestimate of the number of orthopaedic research articles published from LICs.

Another limitation is the fluctuating nature of the classification system utilised by the World Bank, with countries moving between categories each year. Given that this study assessed publications in orthopaedic journals over the last 10 years but used the World Bank Classification for the fiscal year 2018, it only allowed analysis of articles from a single point in time and did not take into account articles from countries that moved into or out of the low-income or lower middle-income categories.

This study also only considered English language literature and more than 400 million Africans (over one third of the continent’s population) live in French- and Portuguese-speaking countries. However, following the initial research of the present study, only one paper was excluded, owing to the fact it was not translated into English (German publication), but we acknowledge this as a limitation.

## Conclusion

The research presented in this paper suggests a lack of orthopaedic research originating from LICs, despite a high proportion of the global burden of disease being located in these countries. We recommend further collaborative initiatives between HICs and LICs as a potential approach to improving the quality and output of orthopaedic research in LICs, helping to guide treatment and improve outcomes. Research collaborations between well-resourced academic institutions with research skills, and clinicians in low-resource settings with high clinical loads and important research questions can be a powerful aspect of global health partnerships – an important message highlighted by The Global Surgery 2030 report by the Lancet Global Commission [11].

## Conflicts of interest

On behalf of all authors, the corresponding author states that there is no conflict of interest.
